# Reasons to Write in Grade 6 and Their Association With Writing Quality

**DOI:** 10.3389/fpsyg.2019.02157

**Published:** 2019-09-24

**Authors:** Renata S. Rocha, Marisa Filipe, Sofia Magalhães, Steve Graham, Teresa Limpo

**Affiliations:** ^1^Faculty of Psychology and Education Sciences, University of Porto, Porto, Portugal; ^2^Mary Lou Fulton Teachers College, Arizona State University, Tempe, AZ, United States

**Keywords:** writing motivation motives to write, attitudes, self-efficacy, opinion essay writing, middle-grade students

## Abstract

Writing is a particularly complex and demanding task that needs to be mastered to assure students’ success at school. In the last decades, the scientific community has been unanimous about the involvement of cognitive and motivational processes in the learning of writing. However, little is still known about some motivation-related processes, such as the reasons why students write. Therefore, this study analyzed the role of motivation in writing in developing writers, by examining the motives to write of 321 sixth graders. We used the Writing Motivation Questionnaire, which is a new instrument tapping the following motivations for writing: curiosity, involvement, grades, competition, social recognition, emotional regulation, and relief from boredom. Findings confirmed the multidimensional nature of motivations to write and supported the validity and reliability of the instrument. Also, results revealed that the strongest motives to write were grades and curiosity, and that curiosity and social recognition were significant predictors of writing quality, above and beyond attitudes and self-efficacy. Together these findings confirm the key role of motivation in writing and provide validity evidence of the Writing Motivation Questionnaire. This seems a useful tool to better understand the motivational processes involved in learning to write. However, despite the increasing research investment in this area, it is still important to carry out further studies that may contribute to the enrichment of the field of writing motivation.

## Introduction

The role of motivation in writing has been acknowledged over the last 30 years. In the 80s, McLeod (1987) discussed the motivational dimension of writing, defining it as “an emotional as well as a cognitive activity” considering that “we feel as well as think when we write” (p. 426). Ten years later, Hayes (1996) put forward a writing model, placing motivation/affect processes (e.g., goals, predispositions, beliefs, attitudes) side by side with cognitive processes. Recently, Graham (2018a, b) highlighted the importance of motivation in writing by proposing the Writer(s)-Within-Community. According to this model, control mechanisms (viz., attention, working memory, and executive control) along with mental and physical operations for producing text (viz., conceptualization, ideation, translation, transcription, and reconceptualization) draw on a set of long-term memory resources. Among these resources, one can find several beliefs that influence writers (e.g., self-efficacy, attitudes, motives to write, value, utility, writing identity, etc.). Providing empirical support to these theoretical proposals, several studies showed that motivation-related variables are associated with writing performance in school-aged (e.g., Graham et al., 2007, 2017; Pajares et al., 2007; Limpo and Alves, 2014, 2017) and undergraduate students (e.g., Baaijen et al., 2014; Sanders-Reio et al., 2014; Limpo, 2018).

This article focuses on the reasons why students write, which have been recently explored in the reading (Schiefele and Schaffner, 2016) and writing domains (Graham et al., 2019; Graham et al., in preparation; Limpo et al., submitted). However, mainly in the writing field, research into the conceptualization of this construct and its predictive value is still at the very beginning. Here, we aimed to test the factorial structure of a new instrument measuring motivations for writing, to determine the strongest motivations in Grade 6, and to examine their contribution to writing quality, above and beyond two other motivation-related predictors known for their association with performance (viz., attitudes toward writing and self-efficacy).

Within the reading domain, Schiefele and Schaffner (2016) proposed a multidimensional conceptualization of reading motivation. This proposal emerged from a comprehensive review of qualitative and quantitative studies examining students motives to read (Schiefele et al., 2012). Authors suggested the following seven dimensions tapping main reasons for reading: to learn more about topics of one’s interest (i.e., curiosity), to experience positive states of feeling, such as getting lost in a story or experiencing imaginative actions (e.g., involvement), to improve one’s grades or achievement in school (i.e., grades), to outperform one’s classmates in school (i.e., competition), to get praise for good performance (i.e., social recognition), to cope with negative emotions, such as anger or sadness (i.e., emotional regulation), and to overcome boredom and to fill in time because other, more preferred activities are not available (relief from boredom). Grounded on the Self-Determination Theory of Deci and Ryan (1985), the dimensions of curiosity, involvement, emotional regulation, and relief from boredom were proposed as components of intrinsic motivation; whereas the dimensions of grades, competition, and social recognition were assumed as components of extrinsic motivation. Intrinsic motivation can be defined as the willingness to engage in an activity for its own sake (e.g., increasing knowledge), and extrinsic motivation can be defined as the willingness to engage in an activity for what it brings (e.g., getting a prize).

Based on theoretical considerations, prior empirical findings, and existing scales, these authors proposed the Reading Motivation Questionnaire (RMQ). This questionnaire is composed of 34 items organized in the seven, above-described dimensions and it was tested in two samples of about 400 German students in Grade 6 (Schiefele and Schaffner, 2016). To examine the factorial structure of the RMQ, authors conducted a set of confirmatory factor analyses (CFA) testing alternative models as well as measurement invariance. Findings provided strong support to the proposed seven-factor structure of the RMQ. Moreover, they found acceptable-to-high reliability estimates, with Cronbach’s alphas above 0.76. Moreover, there was evidence of measurement invariance across gender and reading competence, meaning that the instrument was similar across boys and girls, as well as across students with low and high reading competence. Construct validity was further supported by bivariate significant correlations showing that a larger amount of reading was correlated with higher scores on curiosity (0.25), involvement (0.60), competition (0.13), social recognition (0.10), emotional regulation (0.55), and relief from boredom (0.50); a higher reading fluency was associated with higher scores on involvement (0.20), grades (0.26), emotional regulation (0.18), and relief from boredom (0.10); and a greater reading comprehension was related to higher scores on curiosity (0.18), involvement (0.23), emotional regulation (0.17) and relief from boredom (0.10), but to lower scores on grades (−0.08) and social recognition (−0.09).

Based on this instrument measuring motives to read Graham et al. (in preparation) developed and tested a similar motivation questionnaire for the writing domain. For example, the item “*I read because I know that my friends read a lot*” was transformed into the item “*I write because I know that my friends write a lot.*” Similar changes were made for all items. This questionnaire was then tested with a large sample of fourth- and fifth-grade American students. Following a series of CFA, Graham and colleagues found support for the seven-factor structure proposed for the reading domain, even though some items had been removed. The final version of the Writing Motivation Questionnaire (WMQ) was composed of 28 items, with four items per motivational dimension. Providing further validity evidence on this instrument, authors also showed measurement invariance across grade, gender, race, and socio-economic status. The WMQ also predicted students’ performance on a writing assessment administered by the school district and on a State-wide writing assessment administered 6 months later. The moderate-to-high reliability of the seven factors was also demonstrated, as indicated by the following ordinal omega coefficients: 0.78 for curiosity, 0.74 for grades, 0.78 for competition, 0.76 for boredom, 0.74 for involvement, 0.87 for emotional regulation, and 0.66 for social recognition. In a subsequent study in Grades 6–8, Camping et al. (submitted) examined whether there were differences among students’ motives to write. Authors found that for students with English as the first language, the highest scores were obtained for the motivational dimensions of curiosity and grades. However, these were the least reported motivations for writing among former and current English language learners.

The WMQ was recently adapted to Portuguese by Limpo et al. (submitted), through the following four-step procedure: (a) independent translation of the items by two Portuguese native speakers fluent in English, (b) comparison between the two versions and achievement of a single version, (c) administration of the questionnaire to students followed by discussion, and (d) assurance of semantic equivalence between the Portuguese and English versions through back-translation. After achieving the final version authors examined its factorial structure, tested measurement invariance, evaluated the internal consistency of the motivational dimensions, and analyzed their relationship with external correlates. Authors confirmed the seven-factor structure of the WMQ, even though the analysis conducted led them to drop one item per scale, resulting in a 21-item instrument. This study also showed measurement invariance across two independent groups of third graders. Additionally, across both samples, interitem and item-total correlations, as well as factor loadings, were all adequate. Reliability estimates measured through the ordinal omega for Sample A/Sample B were also good: 0.77/0.79 for competition, 0.69/0.80 for curiosity, 0.77/0.74 for emotional regulation, 0.71/0.81 for grades, 0.71/0.64 for involvement, 0.70/0.73 for relief from boredom and 0.70/0.72 for social recognition.

Furthermore, it was found that all motivations for writing were correlated with measures of self-efficacy (0.15 < *r*s < 0.45). Regarding the correlations of the WMQ with story writing, we found that better stories were associated with stronger curiosity- and grade-related reasons. Overall, the more third graders reported to write for think and write about interesting and important topics as well as for having better grades and achievement in school, the better their stories. Despite the potential of the WMQ to understand the relationship between students’ reasons for writing and their ability to write, only the two above-described studies have used this instrument. Therefore, the current study intends to provide further evidence on the validity and reliability of this tool and to shed light on the contribution of motives to write on writers’ performance, after controlling for attitudes and self-efficacy. It is worth mentioning that at least another conceptualization of motives to write has been proposed (De Smedt et al., 2016, 2017, 2018). De Smedt and colleagues developed the Self-Regulation Questionnaire-Writing Motivation, which encompasses two types of motivation: (a) autonomous motivation, in which engagement in writing results from its inherent satisfaction or value, and (b) controlled motivation, in which engagement in writing results from internal (e.g., guilt) or external pressure (e.g., prizes). The current study extends this dichotomic proposal of writing motivation by considering further motives to writing (e.g., curiosity) and by allowing a fine-grained analysis concerning the specificities of different motives within autonomous/intrinsic and controlled/external components of writing motivation.

There are large disparities between conceptualizations of writing attitudes (for a review see Ekholm et al., 2018). Following Graham et al. (2007), this study adopts a definition of attitudes toward writing along a continuum from positive to negative emotional responses to writing. This construct has also been targeted in the literature under the labels of affect (MacArthur et al., 2015; Limpo, 2018), or liking (Bruning et al., 2013). Prior studies reported correlations between attitudes and writing performance in school-aged children (Bruning et al., 2013; Graham et al., 2017, 2019; Yilmaz Soylu et al., 2017). Moreover, Graham et al. (2007) compared three models testing the direction of attitudes-performance link. Results showed that the best fitting model assumed that writing attitudes influenced writing performance, indicating that the more students liked to write, the better their texts were. It is likely that, compared to writers with a negative attitude, those with a positive one (i.e., enjoying writing) may look for opportunities to write and produce texts more often. Also, they may put more energy in the task and in achieving a good writing product.

Self-efficacy refers to students’ perceptions about their ability to successfully learn or perform academic tasks (Bandura, 1997), such as composing a text. Within the field of writing motivation, self-efficacy is perhaps the most studied variable (Pajares, 2003). Recent studies showed the advantages of using multidimensional measures tapping self-efficacy to accomplish specific writing processes (Bruning et al., 2013; Sanders-Reio et al., 2014; Limpo and Alves, 2017). Bruning et al. (2013) found empirical support for a three-factor model comprising self-efficacy for conventions (i.e., translating ideas into linguistic forms and transcribing them into writing), ideation (i.e., generating good ideas for writing and ordering them), and self- regulation (i.e., managing the cognitive, emotional, and behavioral aspects of writing). Students’ perceptions of ability have been repeatedly found to be among the strongest motivation-related predictors of writing performance (Pajares et al., 2007; Limpo and Alves, 2013; Graham et al., 2017). This effect was also generally observed when self-efficacy focused on specific dimensions (such as conventions, ideation, and self-regulation), though there are some mixed findings in the field. For example, some studies found that the best predictor of performance was self-efficacy for conventions (Bruning et al., 2013; Sanders-Reio et al., 2014; Yilmaz Soylu et al., 2017), whereas others found that it was self-efficacy for self-regulation (Limpo and Alves, 2017). Differences in participants’ age and tested models may explain these different findings.

Examining the role of motivation in writing in developing writers is important not only to deepen our knowledge about the processes involved in this challenging task but also to provide additional strategies to promote it. Motivational beliefs are considered as catalysts for learning in a given domain (Alexander, 1998). By assessing students’ motivation in writing and implementing strategies to booster it, teachers can increase students’ interest to participate in writing activities and make them more eager to learn how to write. An enhanced motivation for writing can be an asset for teachers to help their students to develop and use key cognitive writing processes in a goal-directed, conscious, and sustained way. This is the ultimate practical implication of the present study, which was designed with the overall goal of examining the reasons why school-aged children write. Specifically, we indented to move this recently-started line of research testing the WMQ, which is a new instrument tapping the following main motivations for writing: curiosity, involvement, grades, competition, social recognition, emotional regulation, and relief from boredom. Also, we aimed to provide a strong test of the unique contribution of these motivations to predicting writing quality, after controlling for attitudes and self-efficacy. As surveyed before, there is considerable evidence showing that both attitudes toward writing and self-efficacy beliefs are important motivational predictors of writing quality. The following research questions were put forward:

(1) Does the WMQ organize into a valid and reliable seven-factor model representing students’ motives to write?
(2) Are there any differences among students’ motives to write? If yes, which are the most and the least reported writing motivations by sixth graders?
(3) Which students’ motives to write uniquely contribute to writing quality, after controlling for their attitudes toward writing and writing self-efficacy beliefs?

To answer these questions, we used data from about 300 Portuguese students in Grade 6, who were asked to fill in a set of questionnaires measuring attitudes toward writing, self-efficacy for conventions, ideation, and self-regulation, and motivations for writing. Students were additionally asked to write two opinion essays. Based on the previously reviewed findings using the WMQ, we expected to confirm the multidimensional nature of the questionnaire, to find some motives to be stronger than others, and to observe writing quality to be significantly and uniquely influenced by motivations for writing.

## Materials and Methods

### Participants

In this study participated 321 students from 15 classes in Grade 6 (*M* = 11.7 years, *SD* = 0.5, range = 11–14, 176 girls) from two clusters of schools in the North of Portugal. Students’ socio-economic status and school achievement were measured, respectively, via their mother’s educational level (only available for 60% of the sample) and their marks in Portuguese, Mathematics, and Natural Sciences. Mothers’ educational level was as follows: 5% completed Grade 4, 39% completed Grades 5–9, 26% completed high-school, 22% were graduated, and 7% completed a post-graduation course. Student’s average achievement, assessed from 1 (lowest mark) to 5 (highest mark), was 3.39 for Portuguese (*SD* = 0.63), 3.47 for Mathematics (*SD* = 0.97), and 3.82 for Natural Sciences (*SD* = 0.75).

### Procedure

All students were asked to fill in three instruments measuring attitudes, self-efficacy, and motives to write, in classroom groups of about 20–25 students. For the three scales, the experimenter indicated that there were no right or wrong answers and explained the overall procedure. Items were read aloud to students, who completed the instruments simultaneously and one item at a time. Students were additionally asked to produce two opinion essays for 10 min with 1 week apart. The prompts were “Do you think teachers should give students homework every day?” and “Do you think it is good to have many brothers/sisters?” In a previous pilot study, both prompts were judged by four middle-grade teachers as appropriate to students’ age.

### Measures

#### Writing Motivation Questionnaire (WMQ)

This instrument evaluates students’ motives to write (Graham et al., in preparation). The Portuguese version was developed by Limpo et al. (submitted) and it is composed of 21 items organized into seven dimensions (three items per dimension): curiosity (e.g., I write because I like to think about particular topics), involvement (e.g., I write because I like to create a character that I can identify with), grades (e.g., I write in order to get better grades at school), competition (e.g., I write because it is important to me to write better than other students), social recognition (e.g., I write because I know that my friends write a lot), emotional regulation (e.g., I write because it helps me calm down), and relief from boredom (e.g., I write because it helps me pass the time). Respondents are asked to read a set of sentences illustrating possible reasons for them to write in free time and to indicate the extent to which each reason is true for them. All items are answered on a five-point Likert-type scale ranging from 1 (always true) to 5 (never true). For convenience of interpretation, the responses were reverse-coded. Thus, higher scores indicate higher levels of motivation.

#### Attitudes Toward Writing

Students’ attitudes were measured through the 5-item scale used by Graham et al. (2019): *I enjoy writing*; *Writing is fun*; *I like to write at school*; *I like to write at home*; *Writing is a good way to spend my time*. This scale was translated to Portuguese in the present study. Answers are five on a five-point Likert-type scale ranging from 1 (*always true*) to 5 (*never true*). As for the WMQ, we reversed the responses to facilitate interpretation. Thus, higher scores indicate more positive attitudes toward writing. In this study, CFA showed an excellent fit of the data to the one-factor model, χ^2^(5, *N* = 321) = 6.941, *p* = 0.225, CFI = 0.997, RMSEA = 0.035, *P*(rmsea ≤ 0.05) = 0.656, RMSEA 90% CI [0.000;0.080]. Factor loadings were also very good, ranging from 0.77 to 0.89. Internal consistency measured through the omega assuming ordinal level was 0.93.

#### Self-Efficacy for Writing

Students’ self-efficacy beliefs were measured with the Self-Efficacy for Writing Scale developed by Bruning et al. (2013), validated to the Portuguese language by Limpo and Alves (2018). The scale has 16 items measuring students’ confidence about being able to accomplish specific writing processes in three domains: conventions (e.g., *I can spell my words correctly*), ideation (e.g., *I can think of many ideas for my writing*), and self-regulation (e.g., *I can focus on my writing for at least 1 h*). Answers are given in a scale ranging from 0 (*no chance*) to 100 (*completely certain*), with higher scores indicating higher self-efficacy. In the current study, CFA revealed a good fit of the data to this three-factor model, χ^2^(101, *N* = 321) = 171.661, *p* < 0.001, CFI = 0.942, RMSEA = 0.047, *P*(rmsea ≤ 0.05) = 0.724, RMSEA 90% CI [0.038; 055]. Factor loadings ranged from 0.54 to 0.80 for the conventions sub-scale, from 0.71 to 0.81 for the ideation sub-scale, and from 0.63 to 0.75 for the self-regulation sub-scale. We also found high levels of internal consistency measured through the omega coefficient, assuming interval level (0.80, 0.87, and 0.85 for the conventions, ideation, and self-regulation sub-scales, respectively).

### Writing Quality

One pair of research assistants, blind to study purposes, assessed the quality of students’ texts with a holistic scale based on Cooper (1997). Both judges were asked to evaluate each text with a single score ranging from 1 (*low quality*) to 7 (*high quality*). This score should consider to the same extent the following factors: creativity (i.e., originality and relevance of the ideas), coherence (i.e., clarity and organization of the text), syntax (i.e., syntactic correctness and diversity of the sentences), and vocabulary (i.e., diversity, interest, and proper word usage). To avoid transcription biases on quality assessments, all texts were typed and corrected for spelling errors (Berninger and Swanson, 1994). Several prior studies showed the validity of this procedure to assess text quality across different genres and grade levels (e.g., Harris et al., 2006; Limpo and Alves, 2018). The inter-judge agreement was high, as indicated by the intraclass correlation coefficients for average measures: 0.91 and 0.92 for Text 1 and Text 2, respectively. Thus, the final score for both texts was the average across judges.

### Data Analysis

#### Research Question 1

To analyze the factorial structure of the WMQ, we conducted a CFA using the R system for statistical computing (R Development Core Team, 2005). Since data collection occurred in classroom groups (15 classes), analyses were conducted using the lavaan.survey package, which allows structural equation modeling analyses of clustered data (Oberski, 2014). The method of estimation was maximum-likelihood with robust standard errors, which takes into account the non-independence of the observations and any effects of non-normality. Latent variables were scaled by imposing unit of loading identification constraints. Specifically, the variance of all latent factors was constrained to equal 1.0, so that all factor loadings could be freely estimated. To evaluate model fit we used the chi-square statistic (χ^2^), the confirmatory fit index (CFI), and the root-mean-square error of approximation (RMSEA). CFI values > 0.95 and 0.90, and RMSEA values < 0.06 and 0.10, are considered good and adequate fits, respectively (Hu and Bentler, 1999). This same procedure was followed to run the CFAs for assessing the factorial structure of the attitudes and self-efficacy scales reported in Section “Materials and Methods.”

#### Research Question 2

To test whether there were differences among motives to write we conducted a univariate repeated measures Analysis of Variance (ANOVA) with the WMQ as the repeated measure. The omnibus test was followed-up with pairwise comparisons with Bonferroni correction to determine the most and the least reported motivations for writing by sixth graders.

#### Research Question 3

To examine the contribution of attitudes, self-efficacy, and motives to write to writing quality we used the lavaan.survey package within the R system for statistical computing (R Development Core Team, 2005), as described in Research Question 1 (i.e., taking into account the clustered nature of the data). Specifically, we tested a partially latent structural regression model, in which the dependent variable was a latent factor and the predictors were single indicators. The latent factor was writing quality with two indicators, namely, quality of Text 1 and quality of Text 2. To scale the factor, its variance was constrained to 1.0, so we could freely estimate factor loadings. These latter were 0.72 and 0.63, respectively for Text 1 and Text 2. There were 11 single-indicator predictors, corresponding to the average scores of the attitudes scale, three factors of the self-efficacy (viz., conventions, ideation, and self-regulation), and seven factors of the WMQ (viz., curiosity, involvement, grades, competition, social recognition, emotional regulation, and relief from boredom), assumed to be correlated with each other and to have direct effects on writing quality.

## Results

Before conducting the main analyses, we examined the descriptive statistics and bivariate correlations between all variables (cf. Table 1). In general, we found that attitudes as well as self-efficacy for ideation and for self-regulation were correlated with all motivations for writing; self-efficacy for conventions showed low correlations, albeit low, with grades and curiosity; and all motives to write were moderately associated with each other, with correlations ranging from 0.24 (between emotional regulation and competition) and 0.79 (between involvement and curiosity).

**TABLE 1:** Descriptive statistics and bivariate correlations for all measures.

	**Descriptive statistics**		**Bivariate correlations**											
	***M***	***SD***	**1**	**2**	**3**	**4**	**5**	**6**	**7**	**8**	**9**	**10**	**11**	**12**
(1) Attitudes	2.99	1.06												
**Self-efficacy**														
(2) Conventions	80.41	13.98	0.17^∗∗^											
(3) Ideation	77.46	15.38	0.34^∗∗∗^	0.61^∗∗∗^										
(4) Self-regulation	83.13	23.92	0.38^∗∗∗^	0.56^∗∗∗^	0.68^∗∗∗^									
**Motives to write**														
(5) Competition	2.46	1.13	0.25^∗∗∗^	0.07	0.17^∗∗^	0.23^∗∗∗^								
(6) Curiosity	3.29	1.08	0.61^∗∗∗^	0.16^∗∗^	0.39^∗∗∗^	0.40^∗∗∗^	0.38^∗∗∗^							
(7) Emotional regulation	2.50	1.12	0.65^∗∗∗^	0.08	0.23^∗∗∗^	0.29^∗∗∗^	0.24^∗∗∗^	0.59^∗∗∗^						
(8) Grades	3.60	0.99	0.47^∗∗∗^	0.21^∗∗∗^	0.34^∗∗∗^	0.38^∗∗∗^	0.47^∗∗∗^	0.61^∗∗∗^	0.46^∗∗∗^					
(9) Involvement	3.09	1.01	0.63^∗∗∗^	0.11	0.32^∗∗∗^	0.34^∗∗∗^	0.32^∗∗∗^	0.79^∗∗∗^	0.61^∗∗∗^	0.47^∗∗∗^				
(10) Relief from boredom	2.53	1.06	0.56^∗∗∗^	0.05	0.23^∗∗∗^	0.19^∗∗∗^	0.31^∗∗∗^	0.50^∗∗∗^	0.64^∗∗∗^	0.37^∗∗∗^	0.57^∗∗∗^			
(11) Social recognition	2.50	1.05	0.27^∗∗∗^	0.01	0.16^∗∗^	0.19^∗∗∗^	0.69^∗∗∗^	0.41^∗∗∗^	0.29^∗∗∗^	0.43^∗∗∗^	0.40^∗∗∗^	0.34^∗∗∗^		
**Writing quality**														
(12) Text 1	4.01	0.98	0.22^∗∗∗^	0.34^∗∗∗^	0.29^∗∗∗^	0.24^∗∗∗^	−0.03	0.17^∗∗^	0.11^∗^	0.08	0.10	0.10	−0.12^∗^	
(13) Text 2	3.51	1.02	0.25^∗∗∗^	0.27^∗∗∗^	0.23^∗∗∗^	0.27^∗∗∗^	−0.03	0.19^∗∗∗^	0.12^∗^	0.09	0.14^∗^	0.11^∗^	−0.02	0.45^∗∗∗^

**^∗^p* < 0.05; ^∗^*^∗^p* < 0.01; ^∗∗^*^∗^p* < 0.001.*

### Research Question 1

The first test of the seven-factor model revealed that the covariance matrix of the latent variables was not positive definite. An inspection of this matrix revealed a value of 1.1 between involvement and curiosity, indicating collinearity between these two factors. Based on prior research suggesting higher levels of reliability for the curiosity than the involvement factor (Graham et al., in preparation; Limpo et al., submitted), this latter was removed from the analysis and a six-factor model was tested. Results showed a very good fit of the data to the model, χ^2^(120, *N* = 321) = 222.208, *p* < 0.001, CFI = 0.951, RMSEA = 0.052, P(rmsea ≤ 0.05) = 0.385, RMSEA 90% CI [0.041;0.061]. Factor loadings were also high ranging from 0.59 to 0.84 (cf. Table 2; all items are presented in the Appendix). The analysis of the ordinal omega of each factor revealed high levels of reliability. Values were as follows: 0.86 for Competition, 0.85 for Curiosity, 0.87 for Emotional Regulation, 0.81 for Grades, 0.82 for Relief from Boredom, and 0.80 for Social Recognition. Because De Smedt et al. (2016, 2017, 2018) proposed a dichotomic organization of students’ motives to write, a two-factor model was tested as well (autonomous/intrinsic vs. controlled/extrinsic motivation). Yet, results clearly indicated a poor model fit, χ^2^(153, *N* = 321) = 538.243, *p* < 0.001, CFI = 0.801, RMSEA = 0.110, P(rmsea ≤ 0.05) = 0.001, RMSEA 90% CI [0.100;0.120].

**TABLE 2:** Parameter estimates of the WMQ items by factor.

**Factors and items**	***B***	***SE***	***t***	***b***
Competition				
Item 16	0.97	0.11	8.82	0.76
Item 17	1.06	0.07	15.14	0.78
Item 22	1.02	0.09	11.33	0.77
Curiosity				
Item 6	0.87	0.04	21.75	0.74
Item 20	1.03	0.07	14.71	0.79
Item 23	1.04	0.06	17.33	0.80
Emotional regulation				
Item 8	1.02	0.05	20.40	0.79
Item 13	0.94	0.08	11.75	0.75
Item 18	1.11	0.07	15.86	0.82
Grades				
Item 2	0.86	0.09	9.56	0.72
Item 12	0.95	0.06	15.83	0.84
Item 28	0.80	0.07	11.43	0.60
Relief from boredom				
Item 5	0.86	0.05	17.20	0.64
Item 10	1.06	0.03	35.33	0.82
Item 15	0.87	0.06	14.50	0.72
Social recognition				
Item 14	0.66	0.06	11.00	0.59
Item 24	0.99	0.08	12.38	0.72
Item 26	1.14	0.06	19.00	0.82

*All *p*s < 0.001.*

### Research Question 2

The repeated measures ANOVA showed differences in students’ motives to write, *F*(5,1600) = 124.80, *p* < 0.001, $\eta_{\text{p}}^{2}$ = 0.28. Pairwise comparisons revealed that the strongest motives to write were grades and curiosity, being grade-related motives higher than curiosity-related motives (*t* = 5.91, *p* = 0.001). These two motivations were higher than relief from boredom, social recognition, emotional regulation, and competition (*t*s > 12.12, all *p*s < 0.001). These four motives to write did not differ among each other.

### Research Question 3

As can be seen in Table 3, results showed that together, attitudes, self-efficacy, and motives to write, explained 35% of the variance in writing quality. Attitudes toward writing was a significant predictor of writing quality (*b* = 0.26, *p* = 0.002) as well as self-efficacy for conventions (*b* = 0.34, *p* < 0.001), but not self-efficacy for ideation or self-regulation (*p*s > 0.41). Among the six motives to write, only curiosity (*b* = 0.21, *p* = 0.04) and social recognition (*b* = −0.22, *p* = 0.05) were found to explain unique variance in writing quality. Specifically, text quality was higher for students reporting higher levels of curiosity and lower levels of social recognition.

**TABLE 3:** Parameter estimates in the model regressing writing quality on attitudes, self-efficacy, and motives to write.

**Predictors**	***B***	***SE***	***t***	***p***	***b***
Attitudes	0.30	0.10	3.00	0.002	0.26
Self-efficacy					
Conventions	0.03	0.01	3.00	<0.001	0.34
Ideation	<0.001	0.01	0.30	0.72	0.04
Self-regulation	<0.001	0.01	0.40	0.41	0.09
Motives to write					
Competition	–0.04	0.08	–0.50	0.61	–0.04
Curiosity	0.24	0.12	2.00	0.04	0.21
Emotional regulation	–0.11	0.11	–1.00	0.32	–0.10
Grades	–0.13	0.08	–1.63	0.12	–0.10
Relief from boredom	0.06	0.10	0.60	0.57	0.05
Social recognition	–0.26	0.13	–2.00	0.05	–0.22

## Discussion

This study explored the multidimensional nature of the reasons why sixth graders write and the contribution of these motivations to writing quality. To that purpose, we administered the Portuguese version of the WMQ developed by Limpo et al. (submitted), along with scales measuring writing attitudes and self-efficacy. These variables have been shown to be related to writing performance. Students were additionally asked to write two opinion essays, which were used to assess the quality of their writing. A preliminary analysis showed that, in general, motives to write were related to attitudes and self-efficacy. Although attitudes toward writing were positively associated with all motives to write, findings suggested stronger correlations for intrinsic (0.56 < *r*s < 0.65) rather than extrinsic (0.25 < *r*s < 0.47) motivation components. Concerning self-efficacy, there was a clear pattern suggesting motives to write to be more strongly associated with self-efficacy for ideation and self-regulation than with self-efficacy for conventions. Further research delving into the nature of these relationships seems warranted.

Concerning our main research questions, results revealed that the WMQ was organized in several dimensions tapping motives to write (though the involvement dimension had to be removed), that grades followed by curiosity were the strongest motives to write in Grade 6, and that curiosity and social recognition contributed to writing quality, above and beyond the effects of attitudes toward writing and self-efficacy for conventions. These findings are discussed below in line with the three main research questions underlying the study.

### Research Question: Does the WMQ Organize Into a Valid and Reliable Seven-Factor Model Representing Students’ Motives to Write?

Despite confirming the multidimensional nature of the WMQ, results supported a six- rather than a seven-factor model representing students’ motives to write. When testing the original model, we achieved a not positive definite covariance matrix, which seemed to be signaling a perfect linear dependency between involvement and curiosity. The high correlation between these two dimensions has already been acknowledged in the domain of reading motivation (e.g., Schiefele et al., 2012). Due to this collinearity problem, the involvement dimension – assessing the extent to which students write because this activity allows them to experience positive states of feeling or imaginative actions through writing – was removed from the analysis. Given the reduced number of studies testing the WMQ, we advise caution in interpreting this result. Although we decided to drop the involvement dimension, so that the model could be fitted, future research should continue examining the solution with seven factors.

The six-factor model of writing motivation fitted the data very well. Replicating available research testing the WMQ (Graham et al., in preparation; Limpo et al., submitted), we confirmed the following dimensions: to think and write about interesting topics (i.e., curiosity), to improve one’s grades in school (i.e., grades), to reach higher levels of school achievement than other students (i.e., competition), to get praise for good writing performance (i.e., social recognition), to deal with negative emotions (i.e., emotional regulation), and to fill in time when more preferred activities are unavailable (i.e., relief from boredom). For these six dimensions, factor loadings were all adequate (ranging from 0.59 to 0.84) and the internal consistency was high (0.80 < ordinal ω < 0.87). These findings provide further validity evidence on the Portuguese version of the WMQ.

Moreover, our findings also confirmed the added value of examining different motives to write rather than following a two-factor approach distinguishing between intrinsic/autonomous and extrinsic/controlled motivation as previously proposed (De Smedt et al., 2016, 2017, 2018). On the one hand, CFA results failed to support a two-factor model of the WMQ. On the other hand, as discussed below, the different intrinsic (curiosity, emotional regulation, and relief from boredom) and extrinsic (competition, grades, social recognition) motivations showed distinct and specific links with other motivations and performance variables.

### Research Question 2: Are There Any Differences Among Students’ Motives to Write? If Yes, Which Are the Most and the Least Reported Writing Motivations by Sixth Graders?

The answer to this question is clear-cut: yes, there are differences among students’ motives to write. Specifically, results showed that the strongest motives to write were grades followed by curiosity, and that the weakest motives to write – without differences among them – were relief from boredom, social recognition, emotional regulation, and competition. Differently stated, the most reported reasons for writing were to improve one’s grades in school (a component of extrinsic motivation), and to think and write about interesting topics (a component of intrinsic motivation). These findings replicate those of Camping et al. (submitted). These are relevant results as they seem to indicate that students’ readiness to initiate writing activities is not only dependent upon external rewards that may result from the task, but also upon the satisfying nature of the task itself. Thus, writing might be simultaneously driven by intrinsic and extrinsic incentives. Contrary to our study, Camping et al. (submitted) found significant differences among the other, least reported reasons for writing. In particular, scores for competition and involvement were higher than those for emotional regulation, which in turn surpassed the scores for social recognition and relief from boredom. Understanding the strongest and weakest motives for writing is particularly important in reference to the associations of these reasons with students’ writing quality. This was the goal of the third research question.

### Research Question 3: Which Students’ Motives to Write Uniquely Contribute to Writing Quality, After Controlling for Their Attitudes Toward Writing and Writing Self-Efficacy Beliefs?

The analysis conducted to answer this question provided three main findings worth discussing. First, we found attitudes toward writing to be a significant predictor of writing quality: the more students reported to like writing, the better their texts were. This positive association between attitudes and performance replicates previous studies with school-aged children (Graham et al., 2007, 2017, 2019; Bruning et al., 2013; Yilmaz Soylu et al., 2017). However, as noted by authors of a recent systematic review of writing attitudes, only a handful of studies examined the association of attitudes toward writing with some outcome measure of writing performance (Ekholm et al., 2018). Clearly, more studies addressing this association are needed, with a particular focus on the antecedents of writing attitudes (i.e., which are the main factors underlying children’s positive and/or negative attitudes toward writing?) and on the mechanisms through which attitudes impacts on writing quality (i.e., why is liking to write associated with better texts?).

Second, writing quality was influenced by self-efficacy for conventions, but neither by self-efficacy for ideation nor self-efficacy for self-regulation. These results are in line with prior research, which reported higher associations of self-efficacy for conventions with writing performance compared to the other two dimensions (Bruning et al., 2013; Sanders-Reio et al., 2014; Yilmaz Soylu et al., 2017). The lack of effect of self-efficacy for self-regulation is however contrary to the findings reported by Limpo and Alves (2017) with a sample of Portuguese seventh and eighth graders. We believe these different results might be due not only to the 2-year difference in participants’ average age (11.7 vs. 13.7 years), but also to the models tested. Limpo and Alves tested a path model where self-efficacy and achievement goals were assumed as mediators in the relationship between implicit theories of writing and writing performance. Indeed, the examination of bivariate correlations between the three self-efficacy dimensions and writing quality in that study (0.24 < *r*s < 0.28) were similar to those found in here (0.23 < *r*s < 0.34).

Finally, and more directly related to our research question, we did find a significant effect of motivations to writing quality, above and beyond well-known writing predictors of attitudes and self-efficacy. We found that curiosity had a positive contribution to writing quality and that social recognition had a negative contribution to writing quality. In other words, whereas being motivated to write in order to think more about the topic is associated with better texts, being motivated to write in order to get praise for the good performance is associated with poorer texts. Curiosity and social recognition are specific forms of intrinsic and extrinsic motivation, respectively. A comparison of these findings with those from the reading domain revealed a similar pattern. In their syntheses of research findings of last two decades, Schiefele et al. (2012, p. 453) concluded that “the reviewed studies consistently confirm that intrinsic reading motivation is moderately and positively related to measures of reading competence. In contrast, extrinsic reading motivation was found to be either negatively or not significantly associated with reading competence.” Also in the reading domain, evidence was found that the amount of reading fully mediated the positive effects of intrinsic motivations on reading comprehension; and partially mediated the negative effects of extrinsic motivations. Similar studies in the writing domain are lacking. Further research is needed to shed light on the mechanisms underlying the positive and negative association of curiosity and social recognition with writing quality. Stemming from the reading field, a putative mediator could be writing frequency. Intrinsic motivations such as curiosity may lead individuals to write more. Frequent writing may boost competence by increasing knowledge of and fluency in the enactment of writing processes.

### Limitations and Future Directions

When interpreting the findings of the current study four limitations should be considered along with means to overcome them through possible avenues for future research. First, the WMQ was only administered on one occasion thereby preventing us to test the temporal stability of students’ motives to writing. In the future, it would be worthwhile to administer the instrument over different time intervals. This would provide relevant information not only about the psychometric properties of the WMQ but also about the development of writing motivation across schooling. Given that this study only targeted Portuguese students with an average age of 11 years, additional tests across varying age groups, levels of writing competence, and socioeconomic statuses would be valuable as well.

Second, the relationship between motives to write and writing quality was examined at a single time point with concurrent data collections. Consequently, any developmental conclusion or causal inference grounded in the current findings is unwarranted. An interesting avenue for future research would be the implementation of studies examining the longitudinal impact of students’ motives to write on the development of their writing skills. Such longitudinal findings would allow for a deeper understanding of the motivational mechanisms underlying writing development and provide useful hints to design interventions for promoting motivation in writing.

Third, due to practical reasons, we were not able to administer additional measures, which precluded us to assess students’ frequency of writing. In order to deepen knowledge on the link between students’ motives to write and the quality of their writing, future research should measure how frequently students write across different contexts (e.g., school, home, etc.). As noted before, writing frequency seems a likely mediator in the relationship between writing motivation and writing quality.

Fourth, in addition to the WMQ, the other motivation variables included in this study were attitudes toward writing and self-efficacy for conventions, ideation, and self-regulation. Not only to provide further validity evidence on the WMQ but also to deepen knowledge about the relationship between motivational variables and writing quality, the inclusion of other motivation-related constructs is needed in future research. It would be particularly insightful to test the discriminant validity between motives to write and achievement goals. These latter refer to students’ purposes or desired outcomes for engaging in academic activities (Pintrich, 2000). There is consensus on a trichotomous model of achievement goals, including mastery goals, performance-approach goals, and performance-avoidance goals. Prior correlational research already provided evidence on the benefits of holding mastery goals to produce good writing and their association with self-efficacy beliefs (Limpo and Alves, 2017; Yilmaz Soylu et al., 2017). Additional studies are needed to inform on how motives to write and achievement goals are related to each other and may interact to influence student’s performance in writing.

### Educational Implications

In addition to targeting cognitive-related processes, which has been proved to be an effective way to promote writing quality (Graham and Perin, 2007; Graham et al., 2012), there is the need of taking into account the role of motivation-related processes in the teaching of writing. This claim has been receiving more and more theoretical and empirical support (Bruning and Horn, 2000; Hidi and Boscolo, 2007; Graham, 2018a, b). However, despite current efforts in including motivation as one of the core targets of writing instruction, there is a reduced number of intervention studies testing the added value of instructional activities to increase students’ writing motivation. Several practices, which can be easily included in writing interventions, have been proposed as catalysts of student’s motivation (Ames, 1992; Mueller and Dweck, 1998; Urdan and Schoenfelder, 2006). For example, teachers can propose challenging and meaningful assignments, provide frequent opportunities for success, emphasize the process of learning, stress self-improvement over social comparisons, give regular progress feedback, praise for effort rather than for ability, and promote students’ sense of autonomy. Some of these instructional features are key components of effective teaching models, such as the Self-Regulated Strategy Development model (Harris and Graham, 2009). Together with the growing body of research showing the importance of motivation to produce good writing, this article intends to stimulate the incorporation of these effective motivation-focused practices in the classroom.

## Data Availability Statement

The raw data supporting the conclusions of this manuscript will be made available by the authors, without undue reservation, to any qualified researcher.

## Ethics Statement

The studies involving human participants were reviewed and approved by Ethics Committee of Faculty of Psychology and Education Sciences of the University of Porto. Written informed consent to participate in this study was provided by the participants’ legal guardian/next of kin.

## Author Contributions

TL and SG designed the study. RR, MF, and SM collected and coded the data. TL analyzed and interpreted the data. All authors wrote and reviewed the manuscript and approved its final version.

## Conflict of Interest

The authors declare that the research was conducted in the absence of any commercial or financial relationships that could be construed as a potential conflict of interest.

## Appendix

Items of the writing motivation questionnaire organized by factor.

**Table:**

**Factors and items**
**Competition**
(16) I write because it is important to me to write better than other students.
(17) I write because it is important to me to be among the best students.
(22) I write because it helps me perform better in school than my classmates.
**Curiosity**
(6) I write because I like to think about particular topics.
(20) I write because I can write about topics interesting to me.
(23) I write because I can write about topics important to me.
**Emotional regulation**
(8) I write because it cheers me up when I am in a bad mood.
(13) I write because it helps me calm down.
(18) I write because it makes me feel better.
**Grades**
(2) I write in order to get better grades at school.
(12) I write because it helps me perform well in school.
(28) I write because it is important to how well I do at school.
**Relief from boredom**
(5) I write in order to avoid being bored.
(10) I write because it helps me pass the time.
(15) I write in order to have something to do.
**Social recognition**
(14) I write because I know that my friends write a lot.
(24) I write because one gets praise for writing well.
(26) I write because I like it when other people think I am a good writer.
